# Dentin-Derived Inorganic Minerals Promote the Osteogenesis of Bone Marrow-Derived Mesenchymal Stem Cells: Potential Applications for Bone Regeneration

**DOI:** 10.1155/2020/8889731

**Published:** 2020-11-19

**Authors:** Gang Lei, Yanqiu Wang, Yan Yu, Zehan Li, Jiamin Lu, Xingyun Ge, Na Li, Ana Gloria Cuba Manduca, Jinhua Yu

**Affiliations:** ^1^Department of Geriatric Dentistry, School of Stomatology, Nanjing Medical University, 136 Hanzhong Road, Nanjing, Jiangsu 210029, China; ^2^Key Laboratory of Oral Diseases of Jiangsu Province and Stomatological Institute of Nanjing Medical University, 140 Hanzhong Road, Nanjing, Jiangsu 210029, China; ^3^Endodontic Department, School of Stomatology, Nanjing Medical University, 136 Hanzhong Road, Nanjing, Jiangsu 210029, China

## Abstract

**Background:**

Oral and maxillofacial bone loss is highly prevalent among populations, and nowadays, increased attention has been focused on dentin derivatives serving as desirable graft materials for bone regeneration. In this study, dentin-derived inorganic mineral (DIM) was fabricated with a high-temperature calcination technique and the effects of DIM on the osteogenic differentiation of bone marrow-derived mesenchymal stem cells (BMMSCs) and the bone formation were elucidated.

**Methods:**

The effects of DIM on BMMSC proliferation and apoptosis capacity were evaluated by CCK-8, flow cytometry, and EdU assays. Alkaline phosphatase (ALP) activity detection, ALP staining, alizarin red staining, and osteogenic marker expression analysis were performed to investigate the influence of DIM on the osteogenic differentiation of BMMSCs, as well as the relevant signal mechanisms. The model of critical-sized defects in the calvarium of rats was constructed for exploring the *in vivo* efficiency of DIM on bone regeneration.

**Results:**

Cell viability assays indicated that DIM had no cytotoxicity. BMMSCs cultured with DIM presented a higher level of osteogenic differentiation ability than those in the control group. The activation in ERK and p38 signals was detected in DIM-treated BMMSCs, and both pathways and osteogenic process were suppressed while using ERK inhibitor U0126 and p38 inhibitor SB203580, respectively. Furthermore, the animal experiments revealed that DIM could dramatically enhance new bone formation compared to the control group.

**Conclusion:**

DIM could promote BMMSC osteogenic differentiation via triggering the ERK and p38 MAPK signaling pathways and might be a novel predictable material for facilitating bone formation.

## 1. Introduction

Oral and maxillofacial bone deficiencies due to congenital malformation, tumor resection, trauma, and infection such as periodontitis or periapical inflammation are highly prevalent among populations. Excessive bone loss often results in the spontaneous bone healing failure; thus, valid alternatives to conventional treatments are required for bone repair, such as bone tissue engineering (BTE) [1]. To date, most BTE strategies are aimed at designing a suitable bone graft material for filling the defect volume and providing a supporting substrate by mimicking the extracellular matrix for functional cell migration, proliferation, and differentiation [2]. Bone marrow-derived mesenchymal stem cells (BMMSCs), the nonhematopoietic stem cells located in the bone marrow, are most readily recruited into the defective site and hold the capacity to turn into multiple cell lineages including adipocytes, chondrocytes, and osteoblasts [3–5]. In case of bone injury, BMMSCs respond, remove from bone marrow niche into the peripheral circulation, and migrate through vascular walls into target tissues, and then become the osteoprogenitors that produce osteoblasts [6, 7]. The major superiorities of BMMSCs are their higher accessibility, stronger proliferative capacity, and lower antigenicity, enabling the application of them not only in endogenous regeneration of tissues but also in cell transplantation for tissue engineering [3].

Ideal bone graft materials are the key to success in BTE, which should have the following characteristics: high porosity, noncytotoxicity, osteoconductivity, and ability to promote stem cell differentiation [8]. In the light of the additional surgery and donor site morbidity of autogenous bone, as well as immunological rejection and spread of diseases of allogeneic bone, it is important to develop artificial bone grafts for repairing oral and maxillofacial bone defects [9]. Previous studies have found that tooth and maxillofacial bone both embryologically originate in the neural crest, sharing identical origin [10]. The composition of tooth, especially dentin, made up of 70% minerals, 20% organics, and 10% water, is very close to the bone [11]. Based on these similarities, researchers have considered dentin derivatives as novel bone graft materials for reconstructing oral and maxillofacial bone defects. Demineralization, denaturation, and freeze-dried technique are the conventional preparation methods of dentin [12, 13]. The osteoinduction property of demineralized dentin was first discovered in Urist's report, as evidenced by the process of connective tissues into the bone by endochondral ossification under the inducement [14, 15]. In our previous work, dentin noncollagenous proteins (DNCPs) as the major components of demineralized dentin have been proved to promote BMMSC osteogenic differentiation via the MAPK pathways [16].

Dentin-derived inorganic minerals (DIMs) are prepared from discarded extracted teeth by denaturing dentin at high temperatures. After calcinations, the organic components of teeth are destructed and the remaining powder consists of inorganics; the major ingredients of which are hydroxyapatite (HA) and tricalcium phosphate (TCP) [17]. The two calcium phosphates have been widely employed in clinic for bone reconstruction with excellent biocompatibility, osteoconduction, and similarity to the mineralogical structure of the bone [18]. During the bone repair, HA/TCP composite could be biodegraded and osteoblasts could be absorbed well to promote the reconstruction of the defects [19, 20]. Researches showed that HA/TCP ceramic scaffold could make BMMSCs produce new mineralized extracellular matrix and induce hard tissue formation [21]. It was evident that BMMSCs manifested strong osteogenic potential when cultured with *β*-TCP [22]. Based on these reports, DIM is defined as a material with better osteoconductive property, no immunogenicity, and high porosity which aid in adhesion and survival of cells. Studies performed in ovariectomized rats and patients with jaw bone defects demonstrated that tooth powder and plaster of Paris could be mixed as an effective, yet easily manipulable bone substitute material [17, 23]. In another work, calcined tooth powder in combination with silver nanoparticle additives could promote the periodontal ligament stem cells (PDLSCs) differentiated into odontogenesis [24]. Previously, we used DIM to induce human dental pulp stem cell (DPSC) osteogenic differentiation *in vitro* and have found that the material showed good osteogenic potential [25].

In this research, both *in vitro* cellular experiments and the animal model were conducted for further exploring DIM efficacy on the osteogenic differentiation capacity of BMMSCs before its clinical use.

## 2. Materials and Methods

### 2.1. Material Preparation

This research was approved by the Ethics Committee of the Affiliated Stomatological Hospital of Nanjing Medical University. 80 healthy permanent teeth were collected after obtaining patients' informed consent at the Department of Oral and Maxillofacial Surgery of Affiliated Stomatological Hospital of Nanjing Medical University. First, soft tissues, enamel, and cementum were removed from the collected teeth. Then, successfully separated dentin was calcined using a box furnace at 950°C with a heating rate of 10°C min^−1^ and maintained at 950°C for 30 min. Afterwards, we let the production cool down naturally to room temperature and ground them into powder. The powder was finally filtered out with a 300-mesh screen and collected as dentin-derived inorganic minerals (DIMs). X-ray diffraction (XRD) technique was utilized for determining DIM constituents. 20 g DIM was added into 100 mL alpha minimum essential medium (*α*-MEM, Gibco, Life Technologies, USA) under stirring to form a uniform mixture. The mixture was placed at 37°C in 5% CO_2_ for 5 d and then centrifuged at 400 rpm for 10 min, and the supernatant layer was purified through a 0.22 *μ*m strainer, and finally, was hermetically preserved as a mother solution of DIM-CM. In accordance with the concentration of bioceramic extracts studied in previous studies, the original solution was diluted to 20 mg/mL, 2 mg/mL, 0.2 mg/mL, 20 *μ*g/mL, and 2 *μ*g/mL for the following experiments.

### 2.2. Cell Isolation and Culture

BMMSCs were harvested from 3-week-old male Sprague-Dawley (SD) rats bought from the Animal Core Facility of Nanjing Medical University. In brief, the rats were dissected to separate the femurs and tibias. Then, scissors were used to open the marrow cavity and bone marrow was flushed out with 5 mL syringes supplemented with complete medium into the 15 mL centrifuge tubes. Afterwards, the collection was centrifuged at 1000 rpm for 5 min and subsequently resuspended in *α*-MEM containing 10% fetal bovine serum (FBS, Gibco), 100 g/mL streptomycin, and 100 U/mL penicillin. Cells were then inoculated in culture flask and cultured in an incubator with 5% CO_2_ at 37°C. The medium was changed every three days after the initial plating. When reached 80% confluence, cells were amplified by passage culture.

### 2.3. Alkaline Phosphatase (ALP) Activity and Staining

ALP activity assay kit (Jiancheng, Nanjing, China) was used to detect the ALP activity of treated BMMSCs based on the guidelines provided by the manufacturer. Cell lysis was obtained with 100 *μ*L 1% Triton X-100 for 30 min followed by the ultrasonication and then added into the testing agents. Finally, the ALP activity was examined using a microplate (BioTek, USA). ALP staining was conducted with the NBT/BCIP staining kit (Beyotime, Guangzhou, China). Briefly, the treated BMMSCs were fixed in 4% paraformaldehyde solution for 30 min and then rinsed with PBS twice. Subsequently, cells were stained with ALP premix substrate solution at 37°C for 30 min away from light.

### 2.4. CCK-8 Assay

Cell proliferation was evaluated with the Cell Counting Kit-8 (CCK-8, Dojindo, Kumamoto, Japan) according to the manufacturer's instructions. 1000 cells/well were seeded into 96-well cell culture plates. At a given point in time (1, 3, 5, 7, and 9 d), cells were incubated with 10 *μ*L CCK-8 solution for 2 h away from light. The absorbance at a wavelength of 450 nm was calculated with a microplate reader (BioTek, USA).

### 2.5. 5-Ethynyl-2′-Deoxyuridine (EdU) Assay

Cell proliferation analysis was carried out with an EdU detection kit (RiboBio, Guangzhou, China). Cells were cultured with 50 *μ*M EdU for 2 h at 37°C and then fixed in 4% paraformaldehyde for 30 min and permeabilized with 0.5% Triton X-100 for 10 min. Afterwards, cells were treated with Apollo® reaction cocktail for 30 min. For nuclear staining, cells were treated with Hoechst 33342 for 30 min and visualized with a fluorescent microscope (Olympus, Tokyo, Japan).

### 2.6. Flow Cytometry

Cells were digested by trypsin, resuspended in 4 mL PBS, and centrifuged at 1000 rpm for 12 min. Then, cells were fixed with 75% precooled ethanol at 4°C overnight, followed by staining in 1 mL PI. Cell cycle was detected using flow cytometry (BD Biosciences, San Jose, CA), and proliferation index (PI, *G*_2_/*M* + *S*) in each group was counted and compared. For cell apoptosis detection, cells were digested by EDTA-free trypsin, resuspended in 6 mL PBS and centrifuged at 1000 rpm for 6 min twice, and then resuspended again in binding buffer. Annexin V/PI apoptosis assay kit (BD Pharmingen, Franklin Lakes, USA) was applied to detect cell apoptosis within 15 min in dark. Flow cytometry was employed to calculate the apoptotic rate. In this study, the number of apoptotic cells was defined as the early apoptotic cells (Q2: annexin V+/PI− staining) and the late apoptotic cells (Q4: annexin V+/PI+ staining).

### 2.7. Alizarin Red Staining

Cell mineralization capacity was examined with alizarin red staining. In short, cells were seeded into 12-well plates at a density of 5 × 10^4^ cells per well and incubated for 14 days. Then, cells were fixed in 95% ethanol for 1 h. Subsequently, cells were stained with the alizarin red S solution (pH 4.2, Sigma-Aldrich, USA) for 10 min. The mineralized nodules were observed and photographed under an inverted microscope. For quantification of mineralization, the dye was dissolved using 10% cetylpyridinium chloride (Sigma, UK) for 1 h and then detected by a microplate reader at 540 nm wavelength.

### 2.8. Quantitative Real-Time Polymerase Chain Reaction (qRT-PCR)

Total RNA was isolated from BMMSCs using TRIzol reagent (Invitrogen, Carlsbad, CA, USA) and processed into cDNA with the PrimeScript RT Master Mix Kit (Takara Bio, Otsu, Japan). qRT-PCR was conducted by an ABI 7300 Real-Time PCR System (Applied Biosystems, Carlsbad) by mixing cDNA templates, primers (Sangon Biotech, Nanjing, China), and the SYBR Premix Ex Taq kit (Takara Bio). GAPDH was used as the control. The relative primers are displayed as follows: OCN: forward, 5′-ATTGTGACGAGCTAGCGGAC-3′, reverse, 5′-CTGTGCCGTCCATACTTTCG-3′; OSX: f, 5′-GGAGGCACAAAGAAGCCATA-3′, r, 5′-GGGAAAGGGTGGGTAGTCAT-3′; RUNX2: f, 5′-TTAACGTCAGCAGGAGCAG-3′, r, 5′-CTTCACCCCCAGGACCAAG-3′; ALP: f, 5′-GGAACGGATCTCGGGGTACA-3′, r, 5′-ATGAGTTGGTAAGGCAGGGT-3′; OPN: f, 5′-GCGATCGATAGTGCCGAGAA-3′, r, 5′-TCGTGGCTCTGATGTTCCAG-3′; COL-I: f, 5′-GCAATGCTGAATCGTCCCAC-3′, r, 5′-CAGCACAGGCCCTCAAAAAC-3′; and GAPDH: f, 5′-CACTGAGCATCTCCCTCACAA-3′, r, 5′-GTATTCGAGAGAAGGGAGGGCT-3′.

### 2.9. Western Blot

Total proteins were obtained with RIPA lysis buffer (Beyotime). Individual samples (10 *μ*g/lane) were subjected to sodium dodecyl sulfate-polyacrylamide gel electrophoresis (SDS–PAGE) and blotted onto polyvinylidene fluoride (PVDF, Millipore, MA, USA) membranes. The membranes were blocked using 5% BSA and incubated with primary antibodies, and then incubated with goat anti-rabbit and mouse secondary antibodies (1 : 5000, Proteintech, Wuhan, China) for 1 h. The proteins were visualized with a Western Blotting Imaging System (GE Healthcare, USA). The primary antibodies are displayed as follows: anti-OCN (1 : 1000, ab93876, Abcam, Cambridge, UK), anti-OSX (1 : 1000, ab22552, Abcam), anti-RUNX2 (1 : 1000, ab76956, Abcam), anti-ALP (1 : 1000, ab95462, Abcam), anti-OPN (1 : 1000, ab8448, Abcam), anti-COL-I (1 : 1000, ab34710, Abcam), anti-ERK (1 : 1000, #4695, Cell Signaling Technology, MA, USA), anti-p-ERK (1 : 1000, #4370, CST), anti-JNK (1 : 1000, #9252, CST), anti-p-JNK (1 : 1000, #9255, CST), anti-p38 (1 : 1000, #8690, CST), anti-p-p38 (1 : 1000, #4511, CST), and anti-GAPDH (1 : 1000, AP006, Bioworld, China).

### 2.10. Animal Experiments

The experiments were approved by the protocols of the Institutional Animal Care and Use Committee (IACUC) of Nanjing Medical University (IACUC-1703024). Calvarial defect models were performed in 24 male SD rats at 8 weeks of age bought from the Animal Core Facility of Nanjing Medical University. The rats were given an intraperitoneal injection of pentobarbital to anesthetize them: the dosage was 3.5 mg/100 g body weight. Then, a sagittal incision (length of around 2 cm) was made on the scalp of the rats. Thereafter, the soft tissues covering the calvarium were sharply divided and pushed gently into the lateral. Furthermore, two 5 mm diameter bilateral bone defects were drilled on the calvarias using a portable dental turbine (Xinhe, Wuhan, China) and a circular trephine. Finally, 5 mg DIM powder was subsequently implanted into the bone defect on the left side while the right side was left empty as control. The rats were separated into 3 groups at random: 4-week implantation group, 8-week implantation group, and 12-week implantation group, respectively. Postoperative antibiotic therapy was then provided by intramuscular administration of 2 × 10^5^U/d penicillin for 3 days. The rats were euthanized after 4, 8, and 12 weeks. The calvarias were harvested and analyzed with micro-CT assay and histological analysis.

### 2.11. Microcomputed Tomography (Micro-CT) Assay

After 4, 8, and 12 weeks of surgery, the rats were sacrificed and the calvarias were harvested, followed by fixation in 4% paraformaldehyde, and then examined with a Scanco vivaCT 80 scanner (Scanco Medical AG, Bruttisellen, Switzerland) referring to the following settings: 15.6 *μ*m resolution at 55 kV and 145 *μ*A. Three-dimensional (3D) structures of samples were reconstructed with system software. The bone parameters of the samples including bone mineral density (BMD) and bone volume fraction (bone volume/total volume, BV/TV) were also calculated.

### 2.12. Histological Analysis

Calvarias were fixed in 10% neutral formalin for 48 h and then decalcified in 20% EDTA for 2 months. Thereafter, the specimens were embedded in paraffin. 5 *μ*m thick slices were sectioned and stained with hematoxylin-eosin (H&E) and Masson's trichrome, and then fixed with neutral balsam and analyzed under a microscope (Leica, Wetzlar, Germany).

### 2.13. Statistical Analysis

Each experiment was performed at least in triplicate. The data were presented as the mean ± SD. Statistical analyses were evaluated with SPSS software 16.0 using Student's *t*-test or one-way analysis of variance (ANOVA). *P* values < 0.05 were considered statistically significant.

## 3. Results

### 3.1. 2 mg/mL Is the Optimal Inducement Concentration of DIM

**Figure 1:**
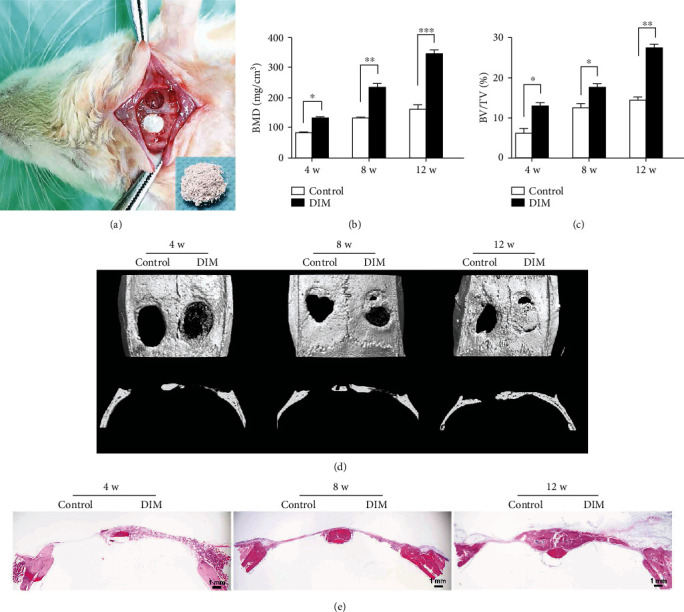
Micro-CT and H&E staining analysis of bone defects treated with DIM at 4, 8, and 12 weeks after surgery. (a) Image of a rat critical-sized calvarial defect model and DIM implantation. (b) Relative 3D reconstruction parameter BV/TV of newly formed bone by micro-CT. ^∗^*P* < 0.05, ^∗∗^*P* < 0.01, and ^∗∗∗^*P* < 0.001. (c) Relative 3D reconstruction parameter BMD of newly formed bone by micro-CT. ^∗^*P* < 0.05 and ^∗∗^*P* < 0.01. (d) 3D reconstruction of calvarial defects and exemplary axial sections by micro-CT. (e) Representative coronal images of calvarial defects by H&E staining. Scalebars = 1mm.

**Figure 2:**
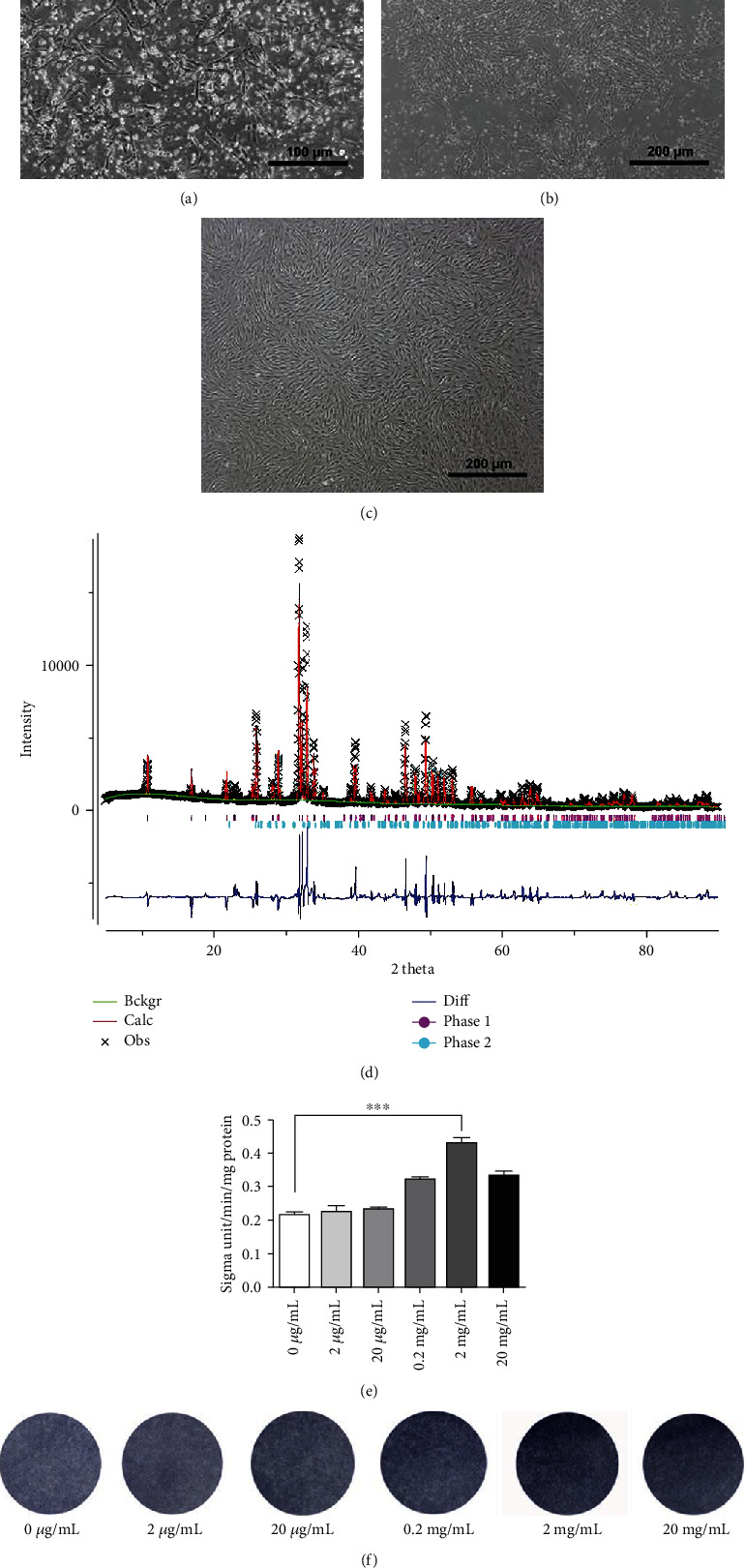
Optimal concentration of DIM for the following experiments. (a, b) Primary BMMSCs. Scalebars = 100*μ*m (a) and 200 *μ*m (b). (c) BMMSCs in passage 3. Scalebars = 200*μ*m. (d) XRD pattern of DIM. (e) ALP activity detection after BMMSCs were treated with 0 *μ*g/mL, 2 *μ*g/mL, 20 *μ*g/mL, 200 *μ*g/mL, 2 mg/mL, and 20 mg/mL DIM on the 3rd day, respectively. ^∗∗∗^*P* < 0.001. (f) ALP staining after BMMSCs were treated with 0 *μ*g/mL, 2 *μ*g/mL, 20 *μ*g/mL, 200 *μ*g/mL, 2 mg/mL, and 20 mg/mL DIM on the 5th day, respectively.

To study DIM effects on the osteogenesis of BMMSCs, we prepared DIM powder and made extracting liquid of that; meanwhile, we isolated and cultured BMMSCs. DIM shaped like white gray powder which was passed through a 300-mesh screen (Figure 1(a)). XRD was conducted to analyze DIM composition. XRD pattern of DIM was exhibited in Figure 2(d). The results showed that DIM was mainly composed of HA and TCP with a ratio of 97.6 : 2.4. The calcium to phosphorus ratio was 1.66 : 1. Primary BMMSCs demonstrated a typical short rod-like or spindle-shaped morphology (Figure 2(a)) and hemocytes decreased in number (Figure 2(b)). BMMSCs in passage 3 were uniform and had the spindle-shaped appearance (Figure 2(c)). The optimal concentration of DIM for inducing BMMSC osteogenic differentiation was determined with ALP activity detection and ALP staining. As illustrated in Figures 2(e) and 2(f), ALP activity and ALP-positive staining cells both increased when BMMSCs were induced with 2 mg/mL DIM, which suggested that 2 mg/mL was the best inducement concentration of DIM.

### 3.2. DIM Has No Effect on BMMSC Proliferation and Apoptosis

**Figure 3:**
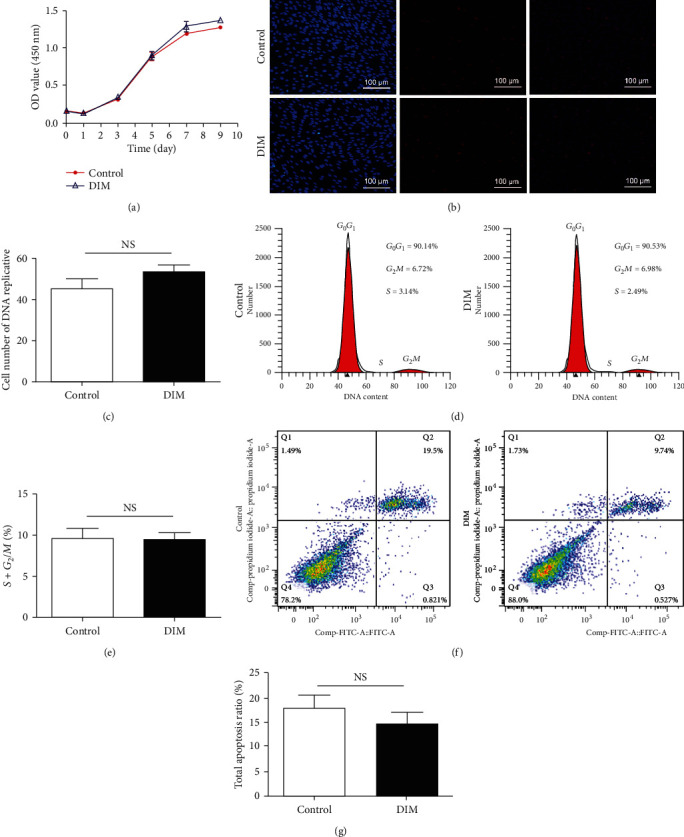
Effects of DIM on BMMSC proliferation and apoptosis. (a) CCK-8 assay was performed to measure the effect of DIM on the proliferation of BMMSCs at the indicated time points. (b) EdU-positive cells under a microscope. Scalebars = 100*μ*m. (c) Quantification of EdU assay. *P* > 0.05. (d) Flow cytometry determination of proportion of BMMSCs in distinct cell cycle phases. (e) Quantitative analysis of (d). *P* > 0.05. (f) Flow cytometry was performed to measure cell apoptosis of BMMSCs treated with DIM. (g) Quantitative analysis of (f). *P* > 0.05.

CCK-8, EdU, and flow cytometry assays were carried out for testing the proliferation of DIM-treated BMMSCs. As shown in Figure 3(a), there was no significant difference in cell proliferation between the DIM group and the control group. Furthermore, the results of EdU assay indicated that the number of EdU-positive cells was same in the DIM group and the control group (Figures 3(b) and 3(c)). Moreover, flow cytometry analysis of the cell cycle distribution showed that there was no significant difference in PI values (*S* + *G*_2_*M*) between DIM-treated BMMSCs (6.98% + 2.49% = 9.47%) and the control group (6.72% + 3.14% = 9.86%) (Figures 3(d) and 3(e)). Taken together, these data suggested that DIM had no effect on BMMSC proliferation. Flow cytometry was also used to evaluate the process of cell apoptosis in the presence of DIM. The results showed that there was no significant difference in cell apoptotic rate (Q2 + Q4) in DIM-treated BMMSCs (9.74% + 88.0% = 97.74%) relative to the control group (19.5% + 78.2% = 97.7%), as represented in Figures 3(f) and 3(g).

### 3.3. DIM Promotes BMMSC Osteogenic Differentiation *In Vitro*

**Figure 4:**
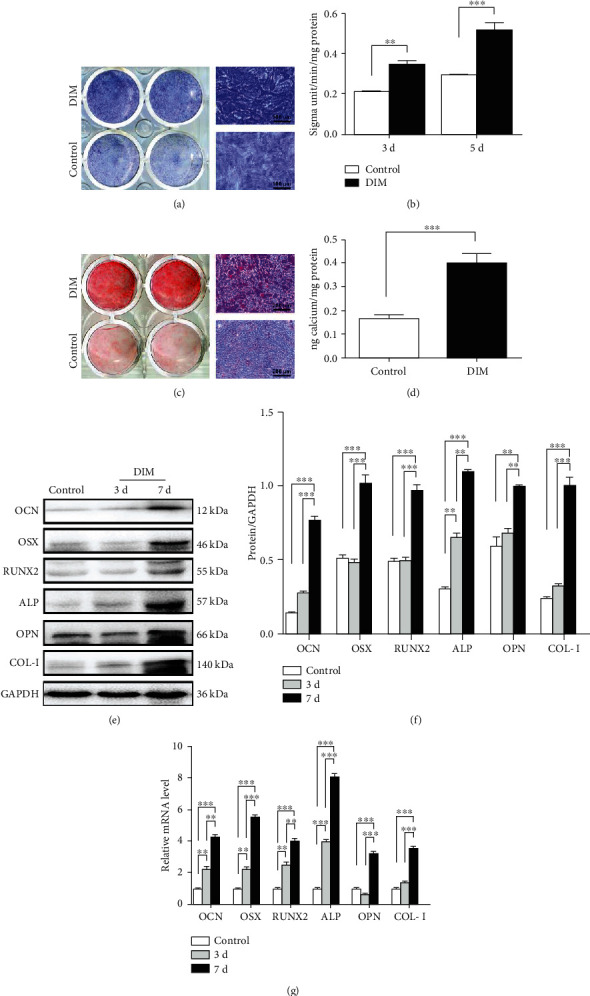
Effects of DIM on BMMSC osteogenic differentiation. (a) ALP staining after BMMSCs were treated with 2 mg/mL DIM on the 5th day. Scalebars = 100*μ*m. (b) ALP activity after BMMSCs were treated with 2 mg/mL DIM on the 3rd and 5th day. ^∗∗^*P* < 0.01 and ^∗∗∗^*P* < 0.001. (c) Alizarin red staining showed calcium deposition after 14 days of culture. Scalebars = 200*μ*m. (d) Quantification of (c). ^∗∗∗^*P* < 0.001. (e) Protein expression levels of OCN, OSX, RUNX2, ALP, OPN, and COL-I were determined with western blot. (f) Quantification of (e). ^∗∗^*P* < 0.01 and ^∗∗∗^*P* < 0.001. (g) mRNA expression levels of OCN, OSX, RUNX2, ALP, OPN, and COL-I were determined with qRT-PCR. ^∗∗^*P* < 0.01 and ^∗∗∗^*P* < 0.001.

To explore the role of DIM on the osteogenic differentiation of BMMSCs, ALP activity detection and ALP staining were first performed as cells were incubated with DIM for 3 or 5 days. As Figure 4(a) showed, compared with the control group, there were more ALP-positive staining cells in the DIM group. Besides, DIM-treated BMMSCs exhibited the higher ALP activity compared to the control (Figure 4(b)). Alizarin red staining was carried out to estimate the mineral deposition of the extracellular matrix produced by BMMSCs. The results showed that DIM led to a notable increase in the number of calcified nodules in comparison with the control group on the 14th day of cell culture (Figures 4(c) and 4(d)). Furthermore, the expression levels of osteogenic markers (OCN, OSX, RUNX2, ALP, OPN, and COL-I) were determined with qRT-PCR and western blot when BMMSCs were incubated with DIM for 3 and 7 days. We found in the presence of DIM, mRNA, and protein expression levels of these markers were elevated in comparison to those in the control group on the 3rd and 7th day, respectively. Moreover, expression levels on the 7th day were obviously higher than those on the 3rd day (Figures 4(e), 4(f), and 4(g)). All the corresponding data showed that DIM could promote BMMSC osteogenic differentiation.

### 3.4. DIM Functions in BMMSC Osteogenic Differentiation via Activating the ERK and p38 MAPK Pathways

**Figure 5:**
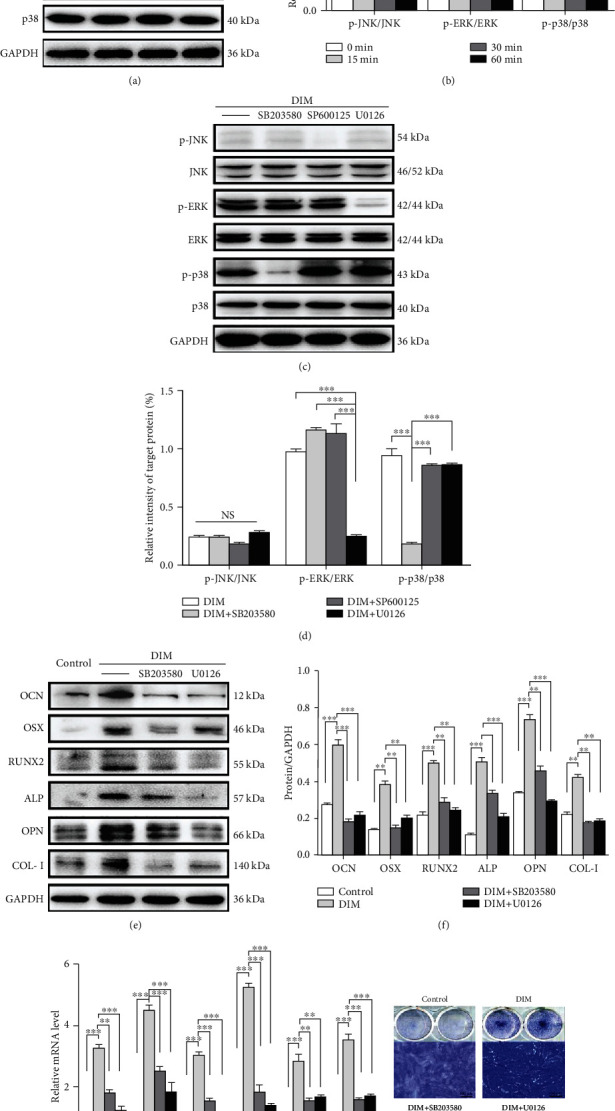
Effects of DIM on the ERK and P38 MAPK pathways involved in BMMSC osteogenic differentiation. (a) Protein expression levels of ERK, p-ERK, JNK, p-JNK, p38, and p-p38 were determined by western blot. (b) Quantification of (a). ^∗∗∗^*P* < 0.001. (c) After BMMSCs were cultured with pathway inhibitors, protein expression levels of ERK, p-ERK, JNK, p-JNK, p38, and p-p38 were determined by western blot. (d) Quantification of (c). ^∗∗∗^*P* < 0.001. (e) Protein expression levels of OCN, OSX, RUNX2, ALP, OPN, and COL-I were determined by western blot. (f) Quantification of (e). ^∗^*P* < 0.05, ^∗∗^*P* < 0.01, and ^∗∗∗^*P* < 0.001. (g) mRNA expression levels of OCN, OSX, RUNX2, ALP, OPN, and COL-I were determined by qRT-PCR. ^∗∗^*P* < 0.01 and ^∗∗∗^*P* < 0.001. (h) ALP staining on the 5th day. Scalebars = 100*μ*m.

In order to evaluate whether DIM exerted effects on the MAPK pathway, MAPK-associated protein expression was detected with western blot at 0, 15, 30, and 60 min. As displayed in Figure 5(a), p-ERK and p-p38 expression reached its maximum at 15 min, and then gradually declined. p-JNK was nearly not expressed from 0 to 60 min.  Figure 5(b) quantitatively shows that p-ERK/ERK and p-p38/p38 were upregulated at 15 min and then dropped, while there was no remarkable difference in the p-JNK/JNK proportion from 0 to 60 min. When the MAPK pathway inhibitors (ERK inhibitor U0126, JNK inhibitor SP600125, and p38 inhibitor SB203580) were used, respectively, the phosphorylation degree of p-ERK in the DIM+U0126 group as well as p-p38 in the DIM+SB203580 group reduced apparently in comparison to that in the DIM group at 15 min. Meanwhile, the expression level of p-JNK had no change in the DIM+SP600125 group compared to that in the DIM group (Figure 5(c)). Quantitative analysis showed the same tendencies (Figure 5(d)). After DIM-treated BMMSCs were cultured with ERK or p38 inhibitors separately for 7 d, the expression of osteogenic markers (OCN, OSX, RUNX2, ALP, OPN, and COL-I) was downregulated in comparison to the DIM group and had no significant difference as compared with that in the control group, indicating that DIM effects were blocked (Figures 5(e), 5(f), and 5(g)). ALP staining showed that the ALP-positive cells in the DIM group on the 5th day were more than the ones in the DIM+U0126 or DIM+SB203580 group (Figure 5(h)). These data suggested that DIM functioned in BMMSC osteogenic differentiation via activating the ERK and p38 MAPK pathways.

### 3.5. DIM Promotes Osteogenesis *In Vivo*

**Figure 6:**
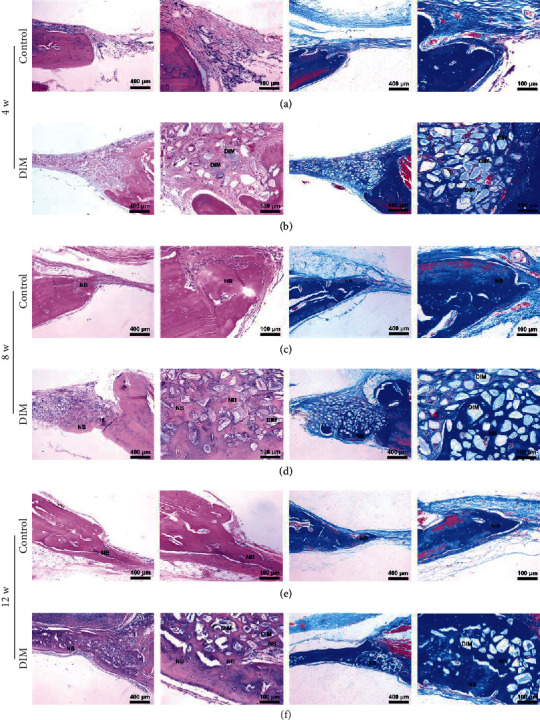
H&E and Masson's trichrome staining analysis of bone defects treated with DIM at 4, 8, and 12 weeks after surgery. (a, b) Coronal images of bone defects in DIM implantation and the control group after 4 weeks. (c, d) Coronal images of bone defects in the DIM implantation group and the control group after 8 weeks. (e, f) Coronal images of bone defects in the DIM implantation group and the control group after 12 weeks. DIM represents the residual material embedded in new bone. NB represents the newly formed bone.

All rats recovered with no complication such as infection and bleeding. The bone regeneration within calvarial defects was observed and analyzed by micro-CT at 4, 8, and 12 weeks after implantation. 3D reconstruction results as shown in Figure 1(d) suggested that at 4 weeks, only small amount of newly formed bone was found in the peripheral part of the bone defect in the DIM group, while there is no bone formation in the control group. At 8 weeks, little new bone was observed in the control group which is much less than that in the DIM group. At 12 weeks, there was a lot of new bone formation growing in the central part of the bone defect in the DIM group and still major bone defects in the control group. Meanwhile, the DIM transplantation was degraded and was not obvious. The higher BV/TV and BMD values of new bone were reached in the DIM group in comparison to the control group at 4, 8, and 12 weeks (Figures 1(b) and 1(c)). Histological analysis of the harvested calvarias from all groups further verified the significant new bone formation in the DIM group, consistent with results of micro-CT. As shown in Figures 6(a) and 6(b), at 4 weeks, the bone defect in the DIM group was mainly filled with residual DIM and plenty fiber network structures of regular array were observed. At 8 weeks, new bone was formed across DIM in bone defects (Figures 6(c) and 6(d)). At 12 weeks, the bone defect treated with DIM was almost completely replaced with reticulated bone and tabular bone (Figures 6(e) and 6(f)).

## 4. Discussion

Repairing and reconstructing oral and maxillofacial bone defects is still full of challenge. Tissue engineering strategies rely mostly on a scaffold composed of osteoconduction and osteoinduction factors, which will allow attachment, growth, and differentiation of MSCs and accelerate regeneration of the bone structure [26]. DIM has been proved to be biocompatible and possesses the osteoconductive property [27]. Su-Gwan et al. [28] have reported that DIM featured good osteoinductivity in animal experiments. Our prior work has shown that DIM could promote the osteogenic differentiation of human DPSCs [25]. However, DIM efficacy on the osteogenesis capacity of BMMSCs has not yet been studied.

In this study, DIM was obtained and we demonstrated that this material induced the *in vitro* osteogenic differentiation of BMMSCs and promoted bone formation in an experimental rat model of calvarial defect. XRD technique was conducted to examine composition characteristics of DIM. The results showed that characteristic peak of DIM is similar with that of HA/TCP, which suggested that DIM mainly consists of HA and TCP. Further analysis revealed that HA to TCP ratio is 97.6 : 2.4 and the calcium to phosphorus ratio is 1.66 : 1, near to that of the human bone. Numerous researches have shown that the ions (such as silicon, calcium, sodium, and phosphorous) play an important role in cell growth and metabolism, also activate some osteogenic-related genes, and promote the mineralization of extracelluar matrix [29]. Calcium channel of lysosomes is necessary in the process of signal transduction during bone remodeling and maintaining bone homeostasis [30]. It has been proved that calcium ions could promote bone formation in a subperiosteal space [31]. Some studies indicate that the mechanisms of calcium phosphate materials in repairing bone defects were based on their released measurable amounts of calcium and phosphorous, which could lead to the apatite reprecipitating onto the material surface and facilitate the bone formation [32]. Rashid et al. have reported that successful bone regeneration with DIM was attributed to localized increased calcium concentration, upregulating the expression level of osteogenic markers (OPN and BMP2) [33].

Generally, to simplify the research work regarding bioceramic materials [34], we made extracting liquid of DIM and confirmed that DIM at a concentration of 2 mg/mL had the optimum inducement capacity for BMMSC osteogenic differentiation, which can be used for further experiments.

As described previously, the early phases in the differentiation of BMMSCs towards osteoblasts occur from day 5 to 14 in incubation and are characterized by ALP expression [35]. The maturation and mineralization of the extracellular matrix produced during osteoblastic differentiation take places from day 14 to 28 in incubation, and alizarin red staining is the most explored parameter for research [36]. While treated with DIM for 5 days, BMMSCs expressed high level of ALP activity compared to the control group. Meanwhile, the amount of ALP-positive staining cells in the DIM group was obviously more than that in the control group. In alizarin red staining, the DIM group presented an upregulation in calcium nodules and the quantification of calcium contents revealed that DIM-treated BMMSCs showed higher amounts of calcium than the control group. Based on these findings, we hypothesized that DIM could promote BMMSC osteogenic differentiation. To further validate the speculation, the expression of related osteogenic markers (OCN, ALP, OPN, and COL-I) and transcription factors (RUNX2 and OSX) was detected. OPN and OCN are both downstream of RUNX2, representing the late stage of the osteogenesis [37]. COL-I, the main organic component of the bone, provides specific functions of scaffold structure for the differentiating cell adhesion [38]. RUNX2 and OSX are vital transcriptional factors associated with the osteoblast differentiation [39]. We found the above markers were highly expressed in DIM-induced BMMSCs on the 3rd and 7th day, as indicated by western blot and qRT-PCR. Therefore, we showed that 2 mg/mL DIM could positively regulate the osteogenic differentiation capacity of BMMSCs.

Mechanisms of DIM in regulating the promotion of BMMSC osteogenic activities needed to be explored. It is well-known that a number of signaling pathways contribute to osteogenic programs of MSCs, such as the mitogen-activated protein kinase (MAPK), Notch, and NF-*κ*B pathways [40]. MAPK superfamily, including extracellular signal-regulated kinase (ERK), c-Jun N-terminal kinase (JNK), and p38, is one of the most thoroughly studied signal transduction systems. It participates in multiple cellular responses to stimulus of inside or outside the body, like proliferation, differentiation, and cell death [41]. ERK and p38 MAPKs play a role in the osteoblast differentiation by directly phosphorylating the master transcriptional factor RUNX2 [42]. In the former exploring studies, we have found ERK and p38 MAPKs are related to the osteogenesis of BMMSCs [16]. Some studies have demonstrated that the biomedical ceramics and their released ions could promote MSC osteogenic differentiation by activating MAPK signals. For example, Xia et al. observed that ERK and p38 MAPKs were activated in the osteogenesis process of BMMSCs while cultured on HA bioceramic scaffolds [43]. In addition, extracellular calcium ions could bond the calcium-sensing receptors (CSR) to start the cascade reactions of the ERK MAPK pathway and induce the bone formation [44]. Calmodulin-dependent protein kinase II (CaMKII)/TAK1 could autophosphorylate in calcium-mediated signal transduction system then activate p38 MAPK to induce cell activities [45, 46]. In this study, the phosphorylation state of the MAPK pathway was tested in BMMSCs when stimulated to differentiate by DIM and we found that ERK and p38 became phosphorylated. While the pathways were treated with their corresponding inhibitors during cell culture with DIM, ERK and p38 were suppressed, and the augmentation of BMMSC osteogenic differentiation capacity (ALP staining and osteogenic marker expression level) was decreased to levels which were not significantly different from the control. Therefore, DIM was proved effective to promote BMMSC osteogenic differentiation by upmodulating osteogenic markers by requiring the activation of ERK and p38 MAPKs. It is speculated that calcium ions released from DIM may determine the activation of ERK and p38 MAPKs by binding CSR or CaMKII.

These *in vitro* experimental results urged us to evaluate the osteogenesis potential of DIM *in vivo*. To assess the bone-repairing ability of graft materials, the natural bone healing in experimental bone defects should be inhibited. The critical-sized defect is known to be a nonunited tissue defect which will never spontaneously heal [47]. In this study, the model of critical-sized defects in calvarium of rats (5 mm in diameter) was established to observe new bone formation after DIM placement [48]. Preferred observation intervals (4, 8, and 12 weeks) were assigned to assess bone formation (4 weeks) and to assess bone maturation, side effects, and absorption of DIM (8, 12 weeks). After the procedure, all rats recovered without immunological rejection and complications such as infection, abnormal bodyweight, and hemorrhage occurred, indicating the implanted material had good biocompatibility with cells. To observe and quantify the state of new bone formation, micro-CT and histological examinations were carried out on the samples at the different time points after surgery. Histological analysis showed that DIM material was intertwined by plenty of reticular fiber-like network structures, and over time, these structures were gradually mineralized and some of them became reticulated bone. At 12 weeks, bulk bones were formed and the fiber-like structures were completely absorbed. Our results suggested that DIM could induce the new bone generation in the critical-sized defects over time; meanwhile, DIM was gradually degraded to provide space for BMMSC differentiation. The better biodegradation than artificial HA brings in favorable clinical prospects for DIM. Combined with the *in vitro* findings, the underlying mechanism of DIM function might rely on the osteoinductive and osteoconductive signals released by DIM. DIM could create a microenvironment in which calcium and phosphorus reached appropriate concentrations to activate the ERK and p38 MAPK pathways, thus induce BMMSC differentiation into osteoblasts, and also serve as a good scaffold material for adhesion, working of cells, and the deposition of newly generated bone. Therefore, it was safe to conclude that the use of DIM did lead to the osteogenesis of BMMSCs and could repair the bone defects in rat critical-sized calvarial defect models. The limitation of the present study is that the precise ingredients of DIM and their direct model of action on MAPKs are not clear and this topic needs our further investigations. Furthermore, the comparison between DIM and other bone graft materials could be analyzed. This study confirmed that DIM was a good bone graft material and may be an alternative therapy for regenerating bone in defects.

## 5. Conclusions

We demonstrated for the first time that DIM could promote the osteogenic differentiation capacity of BMMSCs via activating the ERK and p38 MAPK signaling pathways. In addition, DIM could effectively enhance the bone regeneration as a unique scaffold. Our studies creatively proposed that DIM could be a promising candidate bone graft material capable of mediating efficient bone formation in oral and maxillofacial bone defects.

## Data Availability

The data used to support the findings of this study are included within the article.

## Supplementary Materials

Supplementary Materials Figure 1: ALP staining after BMMSCs were treated with 2 mg/mL DIM on the 3rd day.
