# Supplementary material for: Dentin-Derived Inorganic Minerals Promote the Osteogenesis of Bone Marrow-Derived Mesenchymal Stem Cells: Potential Applications for Bone Regeneration
Source: Stem Cells Int. 2020 Nov 19;2020:8889731. doi: 10.1155/2020/8889731 (PMC7691015; doi:10.1155/2020/8889731)
Supplement: Supplementary Materials — Figure 1: ALP staining after BMMSCs were treated with 2 mg/mL DIM on the 3rd day. [file 8889731.f1.docx]

Supplementary figure:

Figure 1: ALP staining after BMMSCs were treated with 2 mg/mL DIM on the 3rd day.


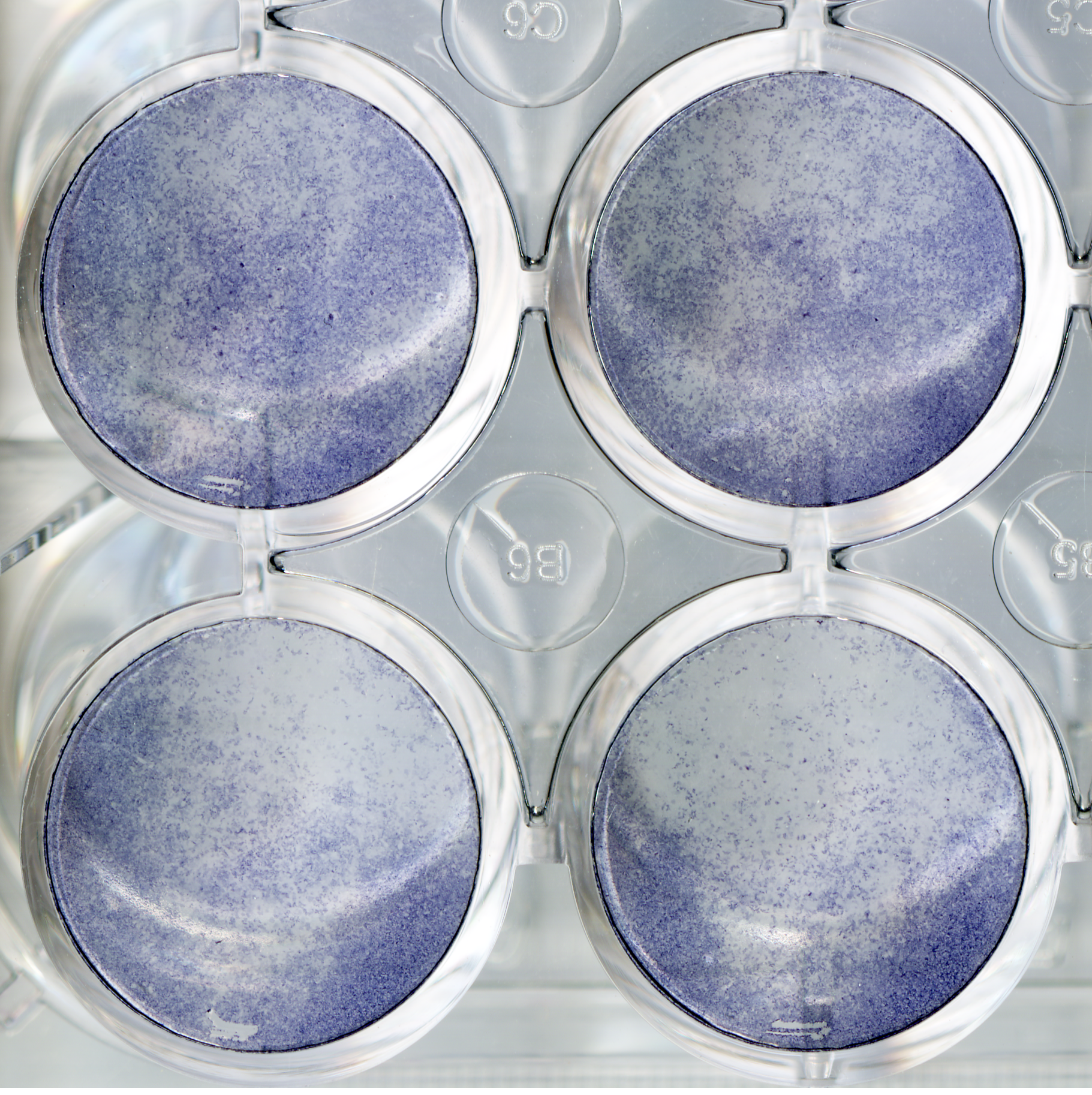


DIM

Control
